# Myocardial Fitness of Bicuspid Aortic Valve Athletes during COVID 19 Pandemic

**DOI:** 10.3390/jfmk7020037

**Published:** 2022-04-29

**Authors:** Melissa Orlandi, Marco Corsi, Vittorio Bini, Luciano De Simone, Laura Stefani

**Affiliations:** 1 Sports Medicine Center, University of Florence, 50121 Florence, Italy; melissa.orlandi@unifi.it (M.O.); marco.corsi1@stud.unifi.it (M.C.); 2 Department of Medicine, University of Perugia, 06123 Perugia, Italy; vittorio.bini@unipg.it; 3 Meyer Hospital, 50139 Florence, Italy; l.desimone@meyer.it

**Keywords:** bicuspid aortic valve, athletes, COVID 19

## Abstract

COVID 19 pandemic has induced a large sedentarism in several kinds of sports. Some peculiar categories of athletes could particularly suffer from a prolonged inactivity as those affected by minimal cardiopathies as bicuspid aortic valve (BAV) athletes. This study aims to verify the myocardial performance in a restricted group of BAV athletes compared to a control group of agonistic athletes evaluated by traditional echocardiography and deformation parameters. 2D standard and deformations parameters were measured at rest conditions in BAV athletes and controls. Particularly EF, LVDD/LVS diameters, GLS rotation and twisting were considered as myocardial performance data; E/A, E1 and A1 as diastolic ones. All the 2D standard parameters measured were within the normal range in both groups, especially the EF value. Significant differences were found in the diastolic function with reduced values of E and E1 waves in BAV vs. controls. The strain analysis showed a significant reduction in GLS measured in 2C, 3C, 4C in BAV if compared to controls, while no significant differences were found in torsional and rotational parameters. These results are suggestive for a potential long term negative impact of inactivity on cardiac performance more evident in BAV athletes, if compared to athletes with normal aortic valve. GLS of LV and RV can be considered as a predictive parameter of this mild dysfunction and assumed as follow-up parameter to restore a progressive training.

## 1. Introduction

The COVID 19 pandemic led to considerable restrictions in the sports field, reducing the hours of daily physical activity and therefore causing a worsening of lifestyle.

Some categories of sport participants under particular surveillance for minimal heart disease may suffer from the negative effects of these restrictions. The forced confinement could potentially induce to a cardiovascular risk factors accumulation and worsening the basic myocardial performance.

Among them, the athletes with bicuspid aortic valve (BAV) and mild valvular dysfunction, eligible for sports activity, are particularly interesting in sport medicine. Previous studies have defined the evaluation of sport fitness criteria and also the specific aspects of cardiac performance [1,2]. In particular, strain analysis as Global Longitudinal Strain (GLS) of the left ventricle (LV) demonstrated to produce a peculiar definition of the cardiac contractility [1,3,4]. However, no data are available about the complete assessment of the LV rotation and torsion, especially in case of long-term inactivity. 

The study aimed to verify, during the period of the COVID19 pandemic, the myocardial performance among athletes with Bicuspid Aortic Valve in absence of regular training and reduced lifestyle level, assessed with echocardiographic evaluation. They have been compared to a group of athletes practicing high intensity sports, equally forced to sedentarism. The study has been planned in order to evaluate the eventual peculiar impact of a prolonged sedentarism on this specific category of subjects in absence of effective aortic valve dysfunction.

## 2. Materials and Methods

A group of 29 BAV athletes (27 males, 2 females, aged 22.9 ± 11.6 years old) and a control group of 16 young athletes practicing rowing (14 males, 2 females, aged 19.3 ± 8.6 years old), similar for intensity of training, were selected for a cardiovascular examination by echocardiographic exam conducted at resting condition. At the time of the investigation, none of the participants received the vaccination against SarsCov2 and they were forced to prolonged sedentarism as a consequence of COVID-19 pandemic. None showed acute cardiovascular or respiratory symptoms.

Among BAV athletes, 5 practiced athletics, 2 basketball, 16 soccer, 3 swimming, 1 cycling and 1 fencing.

Following the ESC guidelines valve classification [5], BAV morphology was type 1 in 3 subjects (anterior-posterior BAV as fusion of the right and left coronary cusp without raphe), type 2 in 18 subjects (anterior-posterior BAV as fusion of the right and left coronary cusp with raphe), type 3 in 5 subjects (fusion of the right and non-coronary cusp, also called right-left BAV), and type 4 in 3 subjects (congenital fusion of the non-coronary and left coronary cusp).

The 2D echo examination was performed by two certified sonographers. It included the standard systo-diastolic and the deformation parameters as global longitudinal strain, basal and apex rotation and torsion of the left ventricular chamber.

Considering the data were from the institutional database, normally obtained during the annual check sport medicine eligibility, in this case the ethical commitee approval is not mandatory. An informed consent was however distributed among the participants. A written informed consent has been obtained from the patients.

### 2.1. Standard 2D Echo Parameters

Following the American Heart Association Guidelines, [6] a complete traditional echocardiogram using My Lab 30 echocardiograph (Esaote, Florence, Italy) equipped with 2.5 MHz probe was performed in each subject at rest. The following parameters were calculated: basal 2D systo-diastolic and Doppler parameters, interventricular septum (IVS), posterior wall thickness (PW), left ventricular end-diastolic diameter (EDD), left ventricular end-systolic diameter (ESD), left atrial dimension (LA) and volume (LAv), aortic root (Aor) dimension, peak velocities of pulsed wave Doppler transmitral flow during early diastole (E) and atrial systole (A), deceleration time of early diastolic flow (DT), and isovolumetric relaxation time (IVRT), with addition of Tissue Doppler (E’, A’, S’) parameters. The evaluation of left ventricular cardiac mass index (g/m^2^) was obtained using the Devereux formula [7]. Ejection fraction (EF) was calculated by the Simpson rule method. The degree of severity of the valvular insufficiency, described as the extent of the regurgitant jet on a 0 to 4 + scale, was assessed using the colour-flow mapping method from the four-chamber view, according to the ACC/AHA Guidelines [8]. Morphology and classification type of bicuspid aortic valve, as typical or atypical shape was detected mainly by shorts axis view and by parasternal long axis view due to the eccentricity closure [5].

### 2.2. Strain Analysis by Speckle Tracking Model

The 2D images of 4-chamber views were post-processed with X-Strain software to provide an angle-independent tool for the evaluation of velocities and strain. This software allows automatic evaluation of the dynamic properties of the endocardial border and of the sub-endocardial tissue from 2D B-mode echocardiographic clips. Strain analysis by speckle-tracking is independent of translational motion, tethering effects of the nearby regions, and it thus allows uniformity of measurements through the normal LV myocardium.

The endocardial border is drawn by the operator in a four-chamber view on a single frame from one annulus to another; the first and last points delineate the mitral plane. The other regional segments points were automatically set by the dedicated software. The LPSS was measured in basal and mid-apical segments of the LW and IVS from the images captured at rest The focused apical 4-chamber view was used for the LV and a modified apical four-chamber view for the RV. In both views frame rates were adjusted to between 40 and 90 frames per second.

During the offline analysis (EchoPac, Version 6.0, GE Healthcare, Horten, Norway) a region of interest was placed around the LV from basal septum through to the basal lateral wall ensuring the whole of the myocardium was encompassed within. This provided six myocardial segments and an average of these provided a global index of LV longitudinal ε. For the RV the Offline analysis involved placing the region of interest around the RV lateral wall only from base to apex. From the short axis view, captured at basal and apex segments of the LV chamber, the rotation was calculated. The net difference of the basal vs. apex rotation values, gave the torsion. Tecnically from that window an as-circular-as-possible short-axis image of the LV apex, just proximal to the level with end-systolic LV luminal obliteration, was obtained by angulation of the transducer. The short-axis views, captured at the mitral valve plane and apex level, were later on processed by the speckle tracking X-Strain software. The software asks the operator to provide the initial position of the tracking points.

### 2.3. Statistical Analysis

All data are reported as mean + SD. The comparisons between LPSS values of basal and mid-apical segments for each group were performed using Student’s unpaired t-test (SPSS Statistics 13, IBM, California State University, Channel Islands, CA, USA). A probability value (*p*) of 0.05 was considered statistically significant. Two-sided 95% exact confidence intervals were used to estimate the difference in the two groups, which is consistent with the two-sided significance level of 0.05.

## 3. Results

45 subjects (29 BAV athletes and 16 rowers) were included in the study. The two groups did not differ for weight, height, BMI and BSA. Instead, there was a significant difference in mean age of the two groups (27.93 ± 11.59 vs. 19.31 ± 8.86 yo; *p* < 0.01). Demographical data are expressed in Table 1.

No significant differences were reported for LV diameters, wall thicknesses and cardiac mass (Table 2); the group with BAV had a significant lower value of E wave (81.07 ± 22.60 vs. 99.06 ± 14.68; *p* = 0.01) and an higher A wave value (55.83 ± 10.59 vs. 42.75 ± 14.71; *p* < 0.001) than rowers, resulting in an E/A ratio lower in BAV group (1.48 ± 0.42 vs. 2.59 ± 1.00; *p* < 0.001). Besides, BAV group presented lower values of E1 (12.55 ± 2.76 vs. 15.53 ± 3.76; *p* < 0.001) but had higher values of A1 (8.76 ± 2.63 vs. 5.6 ± 1.59; *p* < 0.001) and S waves (13.82 ± 4.23 vs. 8.33 ± 1.18; *p* < 0.001). There were no significant differences regarding the aortic measures, with the exception of the diameter of aortic bulb, which was significantly higher in BAV group (33.89 ± 4.71 vs. 28.27 ± 3.28; *p* < 0.001). Evaluating right ventricle echocardiographic parameters, RV area resulted higher in the BAV group (21.78 ± 4.02 vs. 24.8 ± 3.97; *p* = 0.03) while the Rvot, basal and longitudinal diameter values were higher in rowers (26.17 ± 4.02 vs. 21.87 ± 5.32; *p* = 0.03; 27.86 ± 6.85 vs. 35.47 ± 3.78; *p* < 0.001; 65.27 ± 10.37 vs. 72.93 ± 7.69 *p* = 0.02, respectively).

Other significant differences were detected at speckle tracking analysis (Table 3): rowers had all strain values significantly higher than BAV athletes, while no differences were found in torsion and rotational strain parameters. Some parameters resulted significant at t-test analysis (RV area T; GLS4c; RV strain; LV Circ. Basal Strain) were no more significant if adjusted for age at covariance analysis.

## 4. Discussion

BAV is a common congenital heart disease normally compatible with the sports activity [1,2,9]. Prolonged sedentarism can induce a progressive reduction of the myocardial performance either in healthy or in subjects with myocardial disease [9]. This aspect, recently investigated in terms of public health in general population, has not been studied deeply in athletes and particularly in BAV athletes. In the past, a special attention has been adressed in this category especially to those affectd by mild aortic dysfuntion and normally proposed for the eligibility [2]. They normaly train in different kinds of sports with high cardiovascular impact; however, a specific follow up, particularly in presence of prolonged and forced sedentarism, has not been planned. A previous study has considered the importance to evaluate, in sports medicine, a comparison with athletes with normal tricuspid aortic valve by deformation parameters [10]. It has been largely demonstrated that the physical activity and regular sport practice produce benefits on heart’s performance and contribute in maintaining a normal valve function [3]. This aspect has not been however investigated during a particular lifestyle condition and in a population not included in a restricted age range.

A first consideration must be made about the age difference between the two groups: although significant, the difference between mean age of BAV athletes and rowers was about 8 years, which is a relative short amount of time to determine a significant impact on cardiac remodeling, in agreement with what was already described in literature [11,12]. In addition, no relevant influenece of the aortic valvulopaty can be considered to have an impact of the myocardial remodelling in the first line, since all the subjects of the BAV group had a mild regurgitation in absence of stenosis.

A specific attention has been dedicated toward the rotation and twisting. These parameters play a relevant role in the characterization of the heart performance in adaptive [13,14] and maladaptive pathological hypertrophy [15]. It is known that the LV muscle fibres are arranged with a different direction across the myocardium: from a right-handed helix at the endocardium (Endo) to a left-handed helix at the epicardium (Epi) with most intermediate myofibres in approximately circumferential orientation [16]. This geometrical arrangement contributes to a base-to-apex rotational movement whose net difference generates the LV twist. The twisting values found in BAV athletes are normal despite at lower level of the normal range if compared to rowers. A significant difference among these parameters, during the same detraining period, is suggestive for a more evident damage. This aspect could be related in first line to a prolonged sedentarism, especially in the BAV athletes, who might be more sensitive to it. In any case, this aspect will require a more accurate investigation in the specific sector, with dedicated questionnaires and with a more prolonged follow-up involving a larger sample.

Although the evidence of the same and normal LV systo-diastolic diameters and EF in both groups, a potential different micro-structural remodelling of the heart could justify this specific behaviour. No data of the previous echocardiographic condition have been reported in the present investigation, considering the early past of the athletes, was in absolute normal conditions: they had obtained infact the eligibility. No myocardial strain analisys have been made in that time, considering the deformation parameters are not comonly used routinarly. Deformations parameters are more subtle and sensitive in the early detection of the fibres impairment, compared to standard EF evaluation [17,18]. Our data support the evidence of a relevant and significant difference between the two groupsin the LV GLS: this could support, in first line, the hypothesis of potential impact of the mild aortic valve insufficiency among BAV athletes [3], as previously described. On the other hand, considering the normal value of the circumferential contribution of the rotation, another potential component of the subtle reduction of myocardial performance need to be evocated, in particular sedentarism. The circumferential contribution of the rotation and torsion to global heart’s contractility seems to be not influenced by the sedentarism. This aspect could be considered into as normal kind of response of the myocardial contractility in case of athletes.

Despite torsional and rotational parameters reduction appears to go in the same direction of athletes detraining, decreasing values of GLS could be associated to initial cardiac disfunction also in apparently healthy subjects. The data obtained support the importance to perform a complete echocardiographic evaluation in sports medicine, especially after the reduction of the amount of physical activity. Strain analysis can add specific informations on the heart’s function.

## 5. Conclusions

Cardiac function could be negatively affected during a prolonged period of sedentarism or forced reduced physical activity. This specific aspect will need to be more in deep investigated by a long tern follow –up, not applicable in a time of a reduced access to a medical center due to the high level virus infection. Among athletes affected by minor congenital cardiac disease and therefore generally considered within the eligibility, the BAV athletes are a special category under peculiar cardiologic attention long known. The present investigation support the hypothesis to focus on a particular population of athletes normally studied only for their sport performances. Despite regular physical activity plays an important role in general population, our findings suggest that BAV population may particularly benefit from regular training in a more relevant way. Especially, speckle-tracking exam can offer the possibility of detecting subtle contractile impairment: in fact, among strain parameters, GLS was reduced. An eventual role of the moderate intensity of training vs. high intensity in this category of athletes, with particular attention on spontaneous physical activity, will need more investigations in future.

### Limits of the Study

The principal limits of the study is the small size of the sample, justified in any case on the basis of the difficulties to enroll a wide group in the COVID pandemia period when a strong restriction of the physical activity had been made, especially in sport medicine.

An other limit is due to age difference of the two groups investigated that is, in any case, on the basis of the level of training, not in first line considered determinant of the principal aim of the study, addressed to strain analysis that cover a larger age range if compared to the 2D standard echo parameters.

## Figures and Tables

**Table 1:** Demographic characteristics of the participants.

	BAV (*n* = 29)			ROWERS (*n* = 16)			
	MEAN ± SD	CI Inferior	CI Upper	MEAN ± SD	CI Inferior	CI Upper	*p*
Age (y)	27.93 ± 11.59	23.78	32.08	19.31 ± 8.86	9.85	28.78	0.01
Weight (Kg)	73.38 ± 11.19	69.37	77.39	73.13 ± 9.04	37.29	108.96	0.94
Height (m)	1.77 ± 0.07	1.75	1.80	1.78 ± 0.07	0.91	2.66	0.71
BMI (Kg/m^2^)	23.24 ± 2.90	22.20	24.27	22.94 ± 1.84	11.70	34.17	0.71
BSA (m^2^)	1.9 ± 0.16	1.84	1.95	1.90 ± 0.15	0.97	2.83	0.91

Data are expressed as mean ± standard deviation or *n* (%) as appropriate. Boldface = *p* < 0.05.

**Table 2:** Echocardiographic parameters of the participants.

	BAV (*n* = 29)			ROWERS (*n* = 16)				Covariance Analysis Age Adjusted
	MEAN ± SD	CI Inferior	CI Upper	MEAN ± SD	CI Inferior	CI Upper	*p*	*p*
LV IVS (mm)	9.32 ± 0.91	9.00	9.65	9.68 ± 0.68	4.94	14.43	0.18	
LV PW (mm)	8.94 ± 1.08	8.56	9.33	9.49 ± 0.47	4.84	14.14	0.06	
LVEDD (mm)	51.27 ± 3.72	49.93	52.60	50.81 ± 2.97	25.91	75.71	0.68	
LVESD (mm)	31.89 ± 3.53	30.63	33.15	31.5 ± 4.16	16.07	46.94	0.74	
LV Mass (g)	199.22 ± 44.12	183.44	215.01	207.65 ± 29.95	105.90	309.39	0.50	
iLV Mass (g/m^2^)	106.70 ± 17.18	100.55	112.85	109.12 ± 13.49	55.65	162.59	0.63	
EF Simpson (%)	64.83 ± 2.50	63.93	65.72	63.5 ± 5.92	32.39	94.62	0.30	
EF%	64.83 ± 2.50	63.93	65.72	63.5 ± 5.92	32.39	94.62	0.30	
TAPSE (mm)	24.47 ± 3.85	23.09	25.84	23 ± 4.58	11.73	34.27	0.09	
MAPSE (mm)	18.21 ± 1.94	17.52	18.91	18.19 ± 5.01	9.28	27.10	0.98	
E (cm/s)	81.07 ± 22.60	72.98	89.16	99.06 ± 14.68	50.52	147.60	0.01	0.031
A (cm/s)	55.83 ± 10.59	52.04	59.62	42.75 ± 14.71	21.80	63.70	<0.001	0.015
E/A	1.48 ± 0.42	1.33	1.63	2.59 ± 1.00	1.32	3.86	<0.001	<0.001
IVRT (ms)	75.76 ± 9.79	72.26	79.26	72.5 ± 13.62	36.98	108.03	0.36	
DT (ms)	171.97 ± 30.27	161.13	182.80	189.69 ± 43.51	96.74	282.63	0.12	
E1 (cm/s)	12.55 ± 2.76	11.56	13.54	15.53 ± 3.76	7.92	23.14	<0.001	0.043
A1 (cm/s)	8.76 ± 2.63	7.82	9.70	5.6 ± 1.59	2.86	8.34	<0.001	0.002
S (cm/s)	13.82 ± 4.23	12.31	15.34	8.33 ± 1.18	4.25	12.42	<0.001	<0.001
LA Volume (mL)	33.10 ± 3.34	31.91	34.30	33.33 ± 3.68	17.00	49.67	0.84	
RA Volume (mL)	44.05 ± 15.29	38.58	49.52	38.93 ± 15.95	19.85	58.00	0.34	
Aortic Root (mm)	31.72 ± 4.36	30.16	33.28	29.88 ± 2.63	15.24	44.51	0.13	
Aortic Bulb (mm)	33.89 ± 4.71	32.20	35.57	28.27 ± 3.28	14.42	42.12	<0.001	0.003
ST Sinotubular Junction (mm)	31.17 ± 7.97	28.32	34.02	29.6 ± 4.88	15.10	44.10	0.49	
Ascending Aorta (mm)	30.5 ± 9.88	26.97	34.03	30.13 ± 4.55	15.37	44.90	0.89	
RV TDI S’ (cm/s)	14.03 ± 2.72	13.06	15.01	14.07 ± 3.40	7.18	20.97	0.97	
RV (Area T) (mm^2^)	21.78 ± 4.02	20.34	23.22	24.8 ± 3.97	12.65	36.95	0.03	0.060
RV (Area S) (mm^2^)	12.33 ± 2.73	11.35	13.30	13.6 ± 2.77	6.94	20.26	0.17	
Rvot Proximal (mm)	28.12 ± 4.05	26.67	29.57	26.53 ± 4.58	13.53	39.53	0.26	
Rvot Distal (mm)	26.17 ± 4.02	24.73	27.60	21.87 ± 5.32	11.15	32.58	0.01	0.012
RV Basal Diam. (mm)	27.86 ± 6.85	25.41	30.31	35.47 ± 3.78	18.09	52.85	<0.001	<0.001
RV Medium Diam. (mm)	27.59 ± 4.34	26.04	29.14	29.6 ± 3.62	15.10	44.10	0.15	
RV Long. Diam. (mm)	65.27 ± 10.37	61.56	68.98	72.93 ± 7.69	37.20	108.67	0.02	0.017
RV Free Wall (mm)	4.62 ± 0.70	4.37	4.87	4.53 ± 0.73	2.31	6.75	0.72	

Data are expressed as mean ± standard deviation or *n* (%) as appropriate. Boldface = *p* < 0.05.

**Table 3:** Strain parameters of the participants.

	BAV (*n* = 29)			ROWERS (*n* = 16)				Covariance Analysis Age Adjusted
	MEAN ± SD	CI Inferior	CI Upper	MEAN ± SD	CI Inferior	CI Upper	*p*	*p*
4D Global Strain (%)	−17.18 ± 2.64	16.24	18.12	−23.01 ± 3.78	−11.74	−34.29	<0.001	<0.001
GLS4c (%)	−19.52 ± 3.93	18.11	20.93	−20.63 ± 3.45	−10.52	−30.74	<0.001	0.540
GLS3c (%)	−16.83 ± 2.82	15.82	17.84	−20.71 ± 4.08	−10.56	−30.86	<0.001	0.017
GLS2c (%)	−15.91 ± 3.04	14.82	16.99	−21.8 ± 3.15	−11.12	−32.48	<0.001	<0.001
RV Strain (%)	−23.88 ± 4.30	22.34	25.42	−23.01 ± 3.78	−11.74	−34.29	<0.001	0.582
LV Circ. Basal Strain (%)	−22.03 ± 6.37	19.75	24.31	−20.31 ± 2.14	−10.36	−30.27	<0.001	0.510
Torsion (%)	6.34 ± 2.87	5.31	7.36	8.42 ± 2.19	4.29	12.55	0.10	
ROT Apex (%)	4.02 ± 2.75	3.03	5.00	4.93 ± 1.82	2.51	7.35	0.42	
ROT Basal (%)	−4.51 ± 1.56	−5.07	−3.95	−4.72 ± 1.24	−2.41	−7.04	0.77	

Data are expressed as mean ± standard deviation or *n* (%) as appropriate. Boldface = *p* < 0.05.

## Data Availability

Data supporting reported results can be found in archived datasets in Sports Medicine Center-AOUC (Azienda Ospedaliero Universitaria Careggi), University of Florence.
